# eEF2K—a new target in breast cancers with combined inactivation of p53 and PTEN

**DOI:** 10.15252/emmm.201404683

**Published:** 2014-12-03

**Authors:** Hege G Russnes, Carlos Caldas

**Affiliations:** 1 Department of Genetics, Institute for Cancer Research and Department of Pathology, Oslo University HospitalOslo, Norway; 2 K. G. Jebsen Center for Breast Cancer Research, University of OsloOslo, Norway; 3 Cancer Research UK Cambridge Institute and Department of Oncology, Li Ka Shing Centre, University of CambridgeCambridge, UKE-mail: carlos.caldas@cruk.cam.ac.ukDOI 10.15252/emmm.201404683

## Abstract

Extensive efforts have now characterized the somatic molecular alterations in human breast cancer (Cancer Genome Atlas Network, 2012; Stephens *et al*, 2012) and have led to a re-definition of the disease as a constellation of 10 distinct driver-based subtypes (IntClust subtypes) (Curtis *et al*, 2012). The pursuit of druggable targets for each of these subtypes is now pressing. This is elegantly illustrated by the work of Liu *et al* (2014).

See also: **JP Dupuis *et al*** (December 2014)

By studying genetically engineered mouse models, Liu and colleagues found that combined PTEN and p53 inactivation in the mammary gland resulted in claudin-low triple-negative breast cancers [TNBC, a subset of ER-negative breast cancers that are predominantly classified as IC10 and IC4 in Curtis *et al* (2012)], with hyperactive AKT signaling (Liu *et al*, 2014). Drug screening showed these triple-negative cancers to be sensitive to eEF2K-targeted inhibitors. The implications of these findings for human breast cancer are obvious, since both *TP53* and *PTEN* are most commonly mutated in triple-negative breast cancers, although in the TCGA data, combined inactivation of *TP53* and *PTEN* occurs in less than 2.5% of cases (Cancer Genome Atlas Network, 2012).

By using two different transgene mouse model systems, WAP-Cre and MMTV-CreNLST, loss of function of either p53 and/or PTEN was studied with regard to mammary tumor development. The WAP-Cre system allows for secretory epithelium expression. Most of the Cre-mediated recombination is achieved during pregnancy and lactation and preferentially targets alveolar progenitors, while MMTV-CreNLST targets both basal and luminal mammary epithelium progenitors. In both models, the single mutant females developed heterogeneous tumors with little resemblance to human breast cancer. In contrast, double mutants not only developed tumors with a reduced latency, but these tumors frequently exhibited sarcomatoid/spindle cell/mesenchymal features, reflecting characteristics of epithelial mesenchymal transition (EMT), as seen in some triple-negative human breast cancers, also called claudin-low subtype (Prat *et al*, 2010). Gene expression analysis confirmed the observed phenotype, as the majority of the murine PTEN/p53-deficient tumors clustered with claudin-low human breast carcinomas. Interestingly, although both time to tumor development and histological type were similar in the two models, the authors identified several differences by gene expression analysis. Pathways connected to EMT/mesenchymal, UV, stress response and Rb/p53-related cellular senescence were down-regulated in WAP-Cre:PTEN^f/f^:p53^f/f^ tumors but not in MMTV-Cre:PTEN^f/f^:p53^f/f^ tumors, leading to the identification of a 24 differentially expressed genes signature, WCLS (WAP-Cre claudin-low signature). This signature was found associated with survival in patients with claudin-low tumors, although these results will require independent validation.

To address the clinical impact of the combined loss of PTEN and p53 function in human breast cancer, the authors explored larger gene expression data sets. By combining 13 cohorts, data from 2,179 tumors (including 471 TNBC) were analyzed. The identification of the claudin-low subtype, as well as p53 activity and PTEN expression were assessed. The frequency of PTEN-low/p53-low samples was much higher in the TNBC than in other subtypes. Furthermore, among TNBC patients, those with low p53 and PTEN, had a much worse metastatic-free survival than those with active p53 and PTEN signaling. Unfortunately, the data sets analyzed did not have information on *TP53*/*PTEN* gene copy number or mutation status, and so, the significance of the findings is harder to assess. Further, by calculating pathway activation of AKT, PI3K and p53 in the mouse tumors, the AKT pathway was strongly induced in PTEN/p53 deficient tumors. This indicated that loss of PTEN function alone could not deregulate the AKT pathway, a notion that was further strengthened by a strong negative correlation between AKT- and p53-pathway activities in the double PTEN/p53-deficient tumors. In human breast carcinomas, the authors found a weaker correlation between the activities of these pathways. They also found great variability in the level of AKT pathway activity of PTEN/p53-deficient TNBC, which might be due to different cooperating oncogenic networks. Finally, they saw that patients with low p53 but high AKT signaling and/or low PTEN expression had a poorer outcome.

The analyses performed showing the profound impact of concomitant disruption of both PTEN and p53 activity on tumor development, phenotypic subtype and clinical behavior prompted the authors to search for therapeutic targets. The key observations were increased AKT signaling in PTEN/p53-deficient tumors, and the results of the kinome drug screen performed on tumor cultures from one MMTV-Cre:PTEN^f/f^:p53^f/f^ tumor and two human breast cancer cell lines [one basal-like and one claudin-low (Prat *et al*, 2010)] with 238 compounds where the top hit was an inhibitor of eukaryotic Elongation Factor-2 kinase (eEF2K), followed by a c-Jun N-terminal Kinase (JNK) inhibitor. Interestingly, both eEF2K and JNK are implicated in autophagy, and eEF2K seems to be particularly important for survival of tumor cells in hypoxic or nutrition-starved conditions (Leprivier *et al*, 2013). When the authors subjected six different TNBC cell lines to eEF2K inhibitors and measured alterations in gene expression, they found that out of 18 pathways, only AKT signaling significantly correlated with drug sensitivity. Further, both eEF2K and JNK inhibitors suppressed tumor growth of cell line xenografts, reflecting a relationship between p53, PTEN and eEF2K that can be therapeutically targeted (a simplified pathway overview is given in Fig 1). Interestingly, as response to doxorubicin was inversely correlated to AKT pathway activity, the authors tested combinations of eEF2K, JNK and PI3K/mTOR inhibitors with doxorubicin and found both eEF2K and JNK to have additive effects.

**Figure 1:**
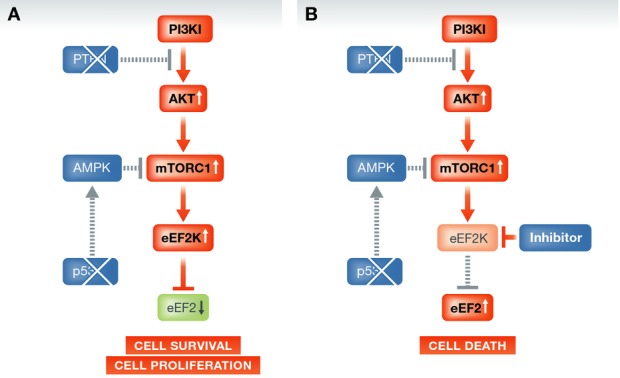
eEF2K, a potential target in PTEN- and p53-deficient breast cancers Low levels of p53 and PTEN can influence cell survival through low eE2F expression (A); inhibition of eEF2K can release this suppression and lead to cell death (B).

This work clearly shows the importance of stratification into tumor subtypes, followed by a focused assessment of key alterations and/or pathways. Targeted treatment requires tailored diagnostics for evaluation of clinical impact in a prospective setting. A clinical trial with an eEF2K inhibitor would need to select patients with TNBC/claudin-low tumors with impaired p53/PTEN activity. There will be several challenges. At the genomic level, mutations in *TP53* have different distribution and clinical relevance across the subtypes reflecting the diversity of p53 function (Silwal-Pandit *et al*, 2014). Likewise, disrupted PTEN function can be due to several mechanisms such as mutations, methylation, localization of the protein and/or modifications (Bassi *et al*, 2013). Thus, in a clinical setting, a combination of genomics (for *TP53*/*PTEN* mutation and copy number assessment) and protein expression (IHC) would be required, at least until robust biomarkers for pathway activity measurements suited for diagnostic laboratories are available.

A stratification of breast cancers into subgroups that enrich for drivers, the ideal therapeutic targets, has been recently robustly validated (Ali *et al*, 2014). It is of major importance to test potential new drugs or combination of drugs in subtype-specific genetically engineered mouse models (GEMMs) (Usary *et al*, 2013), as well as in relevant xenograft models (Hidalgo *et al*, 2014). This, combined with parallel efforts in developing robust molecular diagnostic assays, will be the cornerstone for designing clinical trials of targeted therapeutic approaches, based on knowledge emerged from work such as that reported by Liu *et al* for eEF2K.
